# Integrated Model for Predicting Cancer Therapy-Related Cardiac Dysfunction in Non-Hodgkin Lymphoma

**DOI:** 10.3390/biomedicines13122978

**Published:** 2025-12-04

**Authors:** Daniela Bursacovschi, Oleg Arnaut, Viorica Ochisor, Georgeta Mihalache, Ruslan Baltaga, Vladimir Iacomi, Maria Robu, Valeriu Revenco

**Affiliations:** 1 Department of Heart Failure, Public Healthcare Institution—Institute of Cardiology, MD-2025 Chisinau, Moldova; 2 Department of Human Physiology and Biophysics, Nicolae Testemitanu State University of Medicine and Pharmacy, MD-2004 Chisinau, Moldova; oleg.arnaut@usmf.md; 3 Bioinformatics and Computational Medicine Laboratory, Nicolae Testemitanu State University of Medicine and Pharmacy, MD-2004 Chisinau, Moldova; 4 National Cancer Registry, Public Healthcare Institution—Oncological Institute, MD-2025 Chisinau, Moldova; 5 Department of Cardiology, Nicolae Testemitanu State University of Medicine and Pharmacy, MD-2004 Chisinau, Moldova; 6 Department of Anesthesiology and Intensive Care Nr. 1, Nicolae Testemitanu State University of Medicine and Pharmacy, MD-2004 Chisinau, Moldova; ruslan.baltaga@usmf.md; 7 Public Healthcare Institution—Oncological Institute, MD-2025 Chisinau, Moldova; 8 Department of Pediatrics, Hematology Clinic, Nicolae Testemitanu State University of Medicine and Pharmacy, MD-2004 Chisinau, Moldova; vladimir.iacomi@usmf.md; 9 Department of Hematology, Nicolae Testemitanu State University of Medicine and Pharmacy, MD-2004 Chisinau, Moldova; maria.robu@usmf.md

**Keywords:** chemotherapy-related cardiac dysfunction, heart failure, cancer treatment, prediction model

## Abstract

**Background/Objectives**: Cancer Therapy-Related Cardiac Dysfunction (CTRCD) is a major complication in patients with non-Hodgkin lymphoma (NHL), potentially leading to heart failure and other severe cardiovascular events. Early identification of patients at risk is crucial for timely interventions. **Methods**: A prospective analytical cohort study was conducted on 127 adult NHL patients to evaluate chemotherapy-related cardiac dysfunction over a 6-month period, with the aim of assessing early adverse cardiac effects. Assessments included echocardiography, cardiorespiratory exercise testing, 24-h Holter monitoring, and measurement of cardiac-specific biomarkers (troponin I and NT-proBNP) to identify early subclinical cardiac changes. **Results**: A predictive model for CTRCD was developed using clinical, serological, echocardiographic, cardiopulmonary, and treatment-related parameters in patients with non-Hodgkin lymphoma. The model demonstrated high overall accuracy (94.2%) and strong discriminative ability (AUC 0.95; precision-sensitivity AUC 0.824) for 6-month cardiotoxicity. SHAP analysis identified the most influential predictors as baseline SDNNi, mean daily heart rate, total doxorubicin dose, NT-proBNP, QT corrected interval, hemoglobin, age, left atrial volume, and diastolic function indices (E/e′, E/A). Lower cardiopulmonary reserve was also associated with increased risk. **Conclusions**: The predictive model developed in this study serves as a practical and robust tool for assessing the risk of cancer therapy-related cardiac dysfunction.

## 1. Introduction

Cardiovascular diseases remain a major contributor to mortality among individuals diagnosed with malignant conditions. Contemporary data indicate that the most substantial increase in cardiovascular risk occurs within the first year following the identification of a malignant process [1]. Although the underlying cancer accounts for the majority of deaths in oncology, cardiovascular complications consistently represent the second leading cause of mortality [2]. This pattern has likewise been observed in the therapeutic management of non-Hodgkin lymphoma (NHL). Nevertheless, the timely recognition and early detection of cardiovascular toxicity during NHL treatment continue to pose a significant and persistent clinical concern [3]. Non-Hodgkin lymphomas are neoplasms of lymphoid tissue arising from mature B or T lymphocytes and their precursors, with B-cell-derived subtypes being predominant. NHL represents the most common hematologic malignancy in adults [4]. Following oncologic treatment, a considerable proportion of cancer survivors are exposed to an elevated risk of cardiovascular complications [5,6]. For cardiac injury, cardiomyopathy, and heart failure, current cardio-oncology guidelines recommend the use of the term cancer therapy-related cardiac dysfunction. Despite advances in the treatment of hematologic malignancies, heart failure remains a clinical syndrome characterized by persistent symptoms that impair patients’ quality of life, with a high mortality rate reaching approximately 50% at five years after diagnosis [7]. The incidence of heart failure continues to rise dynamically following treatment regimens including anthracyclines and trastuzumab, reaching approximately 20.1% at five years after therapy. The quality of life of patients with heart failure is closely correlated with New York Heart Association functional class, being superior in those with lower functional class impairment [8].

The early detection of cancer therapy-related cardiac dysfunction facilitates timely initiation of treatment strategies, thereby mitigating the likelihood of developing heart failure and other major cardiovascular complications. Transthoracic echocardiography remains the gold standard for evaluating left ventricular performance, owing to its broad accessibility and cost-efficiency. Moreover, advanced imaging modalities, including tissue Doppler imaging and speckle-tracking echocardiography, allow for the identification of subtle alterations in myocardial mechanics that are not yet evident with conventional assessment techniques [9]. Cardiopulmonary exercise testing provides an integrated assessment of cardiovascular and respiratory function; however, its role in predicting CTRCD has not been fully elucidated. Some studies have reported promising results for CPET-derived parameters, such as peak VO_2_ and the ventilatory efficiency slope (VE/VCO_2_), in the early identification of patients at risk for CTRCD [10]. Within the setting of chemotherapy-associated cardiotoxicity, continuous 24-h Holter electrocardiographic monitoring permits the detection of subtle disturbances in cardiac rhythm and electrical conduction that may precede clinically apparent myocardial dysfunction. Holter recordings are particularly valuable for uncovering silent ischemic episodes and asymptomatic arrhythmic events, thereby aiding in patient-specific risk assessment and guiding individualized therapeutic strategies [11]. However, the precise correlation between rhythm abnormalities identified on Holter monitoring and the subsequent emergence of heart failure or other cardiovascular complications remains to be fully elucidated. Complementary assessment through cardiac biomarkers, including troponins and natriuretic peptides, offers critical insights into subclinical myocardial injury. Elevations in these biochemical markers can reveal early cardiac involvement prior to the manifestation of overt clinical symptoms [12]. Even though a standardized protocol encompassing all these monitoring modalities has not yet been established, the implementation of an integrated surveillance strategy—including periodic echocardiographic assessments, cardiopulmonary exercise stress evaluation, ambulatory ECG monitoring, and evaluation of myocardial injury markers might become an integral part of the clinical protocol for patients undergoing cardiotoxic cancer therapy. This multidisciplinary approach holds the potential to facilitate early detection of CTRCD, enabling prompt interventions and real-time treatment adjustments. Such a strategy has the potential to significantly lower cardiovascular risk while promoting better long-term clinical outcomes for patients with malignancies.

The Heart Failure Association-International Cardio-Oncology Society (HFA-ICOS) risk assessment tool is one of the most widely cited frameworks for CTRCD prediction. It incorporates clinical risk factors, treatment-related variables, and baseline cardiac function to stratify patients into low, medium, and high-risk categories. Although endorsed in guidelines, its discriminative power is moderate and primarily validated in breast cancer populations receiving HER2-targeted therapies [13]. Emerging predictive models for cancer therapy-related cardiac dysfunction include AI-based ECG approaches, machine learning cohort models, and nomogram-based tools. AI-CTRCD models leverage baseline ECGs with transfer learning, achieving AUCs ~0.78, offering early detection and accessibility, but their performance across diverse populations and regimens remains unvalidated and requires high-quality ECG input [14]. Machine learning models using longitudinal clinical, imaging, and laboratory data report AUROC ~0.80, benefiting from multi-modal integration, yet are limited by single-center design and retrospective biases [15]. Nomogram-based models in breast cancer cohorts combining clinical, biomarker, and echocardiographic data show AUCs 0.79–0.82, but external validation is lacking and applicability to other cancers is uncertain [16]. Collectively, these studies highlight the potential of predictive modeling for CTRCD while emphasizing the need for broader validation and adaptable frameworks across cancer types.

## 2. Materials and Methods

The study was prospectively conducted in collaboration between the “Institute of Oncology,” which provided randomly selected patients, and the “Institute of Cardiology” in Chisinau, Republic of Moldova, where they underwent assessment. The study population consisted of 127 adult patients (≥18 years) diagnosed with NHL who provided written informed consent. Enrollment took place between 2022 and 2024, following approval from the Ethics Committee of the State University of Medicine and Pharmacy “Nicolae Testemitanu” (approval no. 7/28 December 2021). The study adhered to the principles of the Declaration of Helsinki. Exclusion criteria included a history of other malignancies, previous oncological therapies (chemotherapy, immunotherapy, or radiotherapy), and underlying cardiovascular disorders, including coronary artery disease, previous myocardial infarction, chronic ischemic heart conditions, various cardiomyopathies, or post-inflammatory/infiltrative myocardial abnormalities. Individuals with moderate to severe valvular heart disease (encompassing at least moderate stenosis or regurgitation) or advanced heart failure (NYHA class III–IV) were likewise excluded from the study. At baseline, patients completed a structured questionnaire regarding cardiovascular risk factors—including diabetes mellitus, hypertension, dyslipidemia, and smoking—as well as comorbidities such as chronic kidney disease, proteinuria, chronic obstructive pulmonary disease, and thyroid disorders. Clinical data regarding lymphoma characteristics and administered treatments, including specific chemotherapy or immunotherapy regimens and cumulative doxorubicin dose at 6 months, were systematically recorded. Participants underwent two comprehensive evaluations: prior to initiation of antitumor therapy and at a 6-month follow-up according to the study design. According to the diagnostic criteria established by the 2022 cardio-oncology guidelines, cancer therapy-related cardiac dysfunction was identified in 18 (14.2%) patients at the 6-month follow-up evaluation [7].

**Figure 1:**
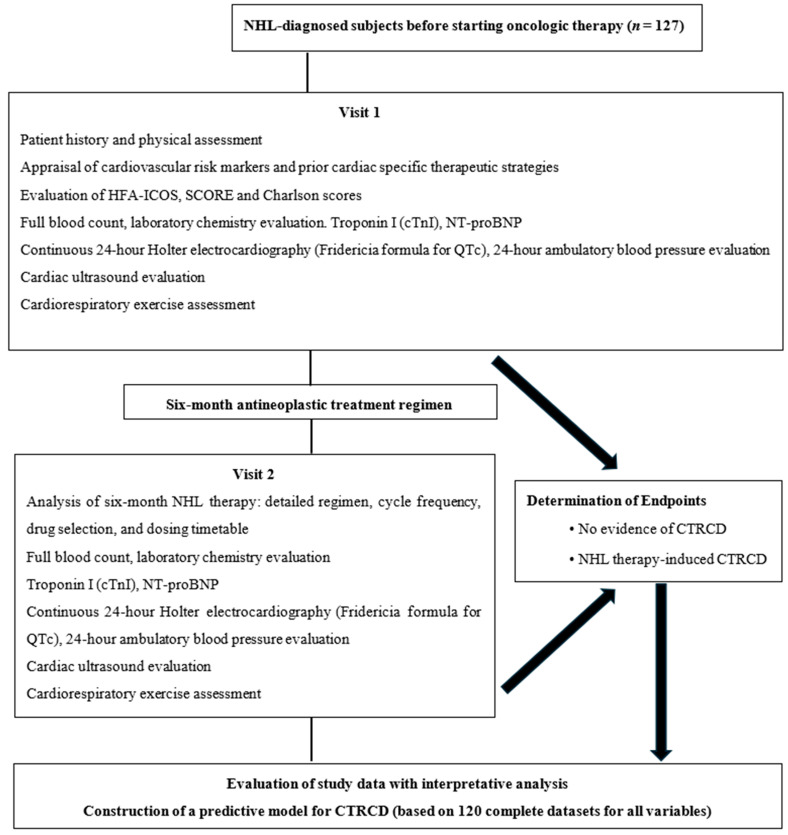
Study design.

Assessments included transthoracic echocardiography, cardiopulmonary exercise testing, and laboratory analyses. Serological measurements focused on troponin I and NT-proBNP levels, obtained both at baseline and after 6 months of therapy. Echocardiographic examinations were performed by a single experienced cardiologist using a General Electric Vivid E95 system (GE VINGMED ULTRASOUND A/S, Horten, Norway). Cardiac chamber dimensions and function were evaluated according to the 2015 recommendations of the American Society of Echocardiography and the European Association of Cardiovascular Imaging [17]. Twenty-four-hour Holter ECG monitoring was performed using the ECGpro Holter system (2021), with automated analysis followed by manual verification for accuracy. Key parameters included mean, minimum, and maximum heart rate, rhythm classification (sinus rhythm, atrial fibrillation), and the total number of supraventricular and ventricular ectopic beats. Episodes of sustained supraventricular or nonsustained ventricular tachycardia were identified based on standard criteria (18). Heart rate variability metrics analyzed included SDNN, SDANN, and SDNNi, reflecting autonomic modulation and myocardial function, with reductions indicating increased cardiovascular risk [18,19]. Corrected QT interval (QTc, Fridericia formula: QTc = QT/∛RR) and PQ intervals were measured continuously, with QTc ≥480 ms or sex-specific intermediate thresholds (men: 450–480 ms; women: 460–480 ms) flagged as potential arrhythmic risk. ST-T segment changes were classified as ischemic if ST depression ≥1 mm persisted ≥1 min but ≤20 min, separated by ≥1 min from other episodes. PQ intervals between 120 and 200 ms were considered normal. Patients were familiarized with the cardiopulmonary exercise testing equipment (Quark CPET, Cosmed, Rome, Italy), utilizing equipment such as a cycle ergometer along with devices for assessing respiratory parameters, with participants directed to perform exercise at maximal intensity, discontinuing if they experienced symptoms like chest discomfort, palpitations, or lightheadedness. CPET was performed according to the 2019 American Thoracic Society and 2020 European Society of Cardiology guidelines for cardiopulmonary exercise testing and exercise in cardiovascular patients [20,21], with continuous 12-lead ECG, blood pressure, and oxygen saturation monitoring. A symptom-limited, incremental protocol was applied, progressing every minute through rest, unloaded cycling, progressive exercise, and recovery phases. Key CPET parameters analyzed included hemodynamics (baseline and peak heart rate, systolic/diastolic blood pressure, oxygen saturation), peak VO_2_, VO_2_ at anaerobic threshold, %VO_2_ at threshold, VO_2_ pulse, VO_2_/work rate, ventilatory efficiency (VE/VCO_2_), minute ventilation, ventilatory reserve, end-tidal CO_2_, and oxygen uptake efficiency slope. A peak VO_2_ < 14 mL/kg/min was considered indicative of reduced aerobic capacity and higher cardiovascular risk, while VE/VCO_2_ >30 reflected ventilatory inefficiency and predicted increased long-term heart failure severity, hospitalizations, and mortality [22]. The study design is presented in Figure 1.

### Statistical Analysis

The main dataset was processed and examined using RStudio (v. 2024.09.1+394, https://www.rstudio.com/) and Python (v. 3.12.3, https://www.python.org/), ensuring that the entire statistical workflow could be fully reproduced. For numerical variables, descriptive statistics were calculated, including minimum, maximum, mean, standard deviation, median, and interquartile range. Differences between groups for numerical data were assessed using the Mann–Whitney U test. For categorical variables, both absolute counts and percentages were calculated, with percentages reported alongside their 95% confidence intervals. Associations between categorical variables were evaluated using Pearson’s Chi-squared test, applying the Monte Carlo method with 100,000 simulations. A significance level of 0.05 (α = 0.05) was used for all statistical tests. The predictive performance of the model for CTRCD was evaluated using the area under the receiver operating characteristic curve. Feature contributions were interpreted with SHAP (SHapley Additive exPlanations) analysis, with descriptive statistics of SHAP values calculated for each feature. Visualizations, including summary and dependence plots, were used to illustrate the relative importance and interactions of the predictors.

## 3. Results

### 3.1. Demographic Profile, Comorbidities, and Treatment Features in Patients with Non-Hodgkin Lymphoma

The study cohort comprised 127 patients diagnosed with non-Hodgkin lymphoma, of whom 72 were men (56.7%, 95% CI: 48–65) and 55 were women (43.3%, 95% CI: 35–52). In terms of place of residence, 66 individuals lived in city settings (52.0%, 95% CI: 43–61), while 61 lived in village settings (48.0%, 95% CI: 39–57). The median body mass index was 25.9 kg/m^2^ (IQR = 7.0), ranging from 18.6 to 48.7 kg/m^2^. According to BMI categories, 59 subjects (46.5%, 95% CI: 38–55) were eutrophic, 37 (29.1%, 95% CI: 21–37) were overweight, 21 (16.5%, 95% CI: 10–23) had class I obesity, 7 (5.5%, 95% CI: 1.5–9.5) had class II obesity, and 3 subjects (2.4%, 95% CI: 0.0–5.0) presented with class III obesity. Eighty-four patients exhibited dyslipidemia (66.1%, 95% CI: 58–74), while metabolic syndrome was documented in 18 subjects (14.2%, 95% CI: 8.1–20). The most frequent concurrent medical condition was arterial hypertension, identified in about 50 individuals (39.3%). Among these, grade II hypertension was the most common, observed in 35 patients (27.6%, 95% CI: 20–35), followed by grade III hypertension in 11 patients (8.7%, 95% CI: 3.8–14) and grade I hypertension in 4 cases (3.1%, 95% CI: 0.11–6.2). A history of chronic obstructive pulmonary disease was present in 27 patients (21.3%, 95% CI: 14–28). Pre-existing chronic kidney disease was documented in 14.9% of patients. According to the KDIGO 2020 classification, 14 patients (11.0%, 95% CI: 5.6–16) had CKD stage G1–G2, and 5 patients (3.9%, 95% CI: 0.55–7.3) had stage G3 disease. No cases of more advanced CKD or other severe decompensated comorbidities were reported. Considering the identified comorbidities and the individual characteristics of the patients, the entire study cohort was assessed using the Charlson Comorbidity Index. Based on this tool, three risk categories were defined: 17 patients (13.4%, 95% CI: 7.5–19) were classified as low risk, reflecting a generally good health status and a relatively low probability of major adverse events or long-term mortality. A substantially larger proportion of 54 patients (42.5%, 95% CI: 34–51) fell into the moderate-risk category, suggesting the presence of concurrent conditions that may influence disease progression. In contrast, 56 individuals (44.1%, 95% CI: 35–53) were identified as high risk, indicating a considerable burden of serious comorbidities that may significantly compromise overall health status and require more intensive management. Cardiovascular risk according to the SCORE algorithm was estimated in line with the most recent 2021 ESC Guidelines on cardiovascular disease prevention. Patients were distributed across three categories: low-to-moderate cardiovascular risk in 13 cases (10.2%, 95% CI: 5.0–16), high cardiovascular risk in 30 cases (23.6%, 95% CI: 16–31), and very high cardiovascular risk in 84 patients (66.1%, 95% CI: 58–74). Subsequently, all patients were assessed using the HFA-ICOS risk stratification model for cardiotoxicity in oncology. According to this model, 44 patients (34.6%, 95% CI: 26–43) were identified as having a low risk of cardiotoxicity, 31 patients (24.4%, 95% CI: 17–32) were classified as moderate risk, and 5 patients (3.9%, 95% CI: 0.55–7.3) were categorized as high or very high risk. Importantly, in 47 patients (37.0%, 95% CI: 29–45), risk assessment could not be performed because they had not received anthracycline-based therapy within their treatment regimen. Table 1 outlines the baseline and clinical profiles of the cohorts with and without CTRCD.

The indolent form represented the predominant subtype, being identified in 78 patients (61.4%, 95% CI: 53–70), and was associated with a more protracted and less aggressive clinical evolution. Precursor B-lymphoblastic lymphoma accounted for the overwhelming majority of diagnoses—123 cases (96.9%, 95% CI: 94–100). In terms of disease staging, stage IV was the most frequently encountered, recorded in 89 individuals (70.1%, 95% CI: 62–78). This was followed by stage III in 18 patients (14.2%, 95% CI: 8.1–20), stage II in 16 cases (12.6%, 95% CI: 6.8–18), while stage I was uncommon, observed in only 4 patients (3.1%, 95% CI: 0.11–6.2). As for the primary tumor site, peripheral lymph node involvement predominated (94 patients, 74.1%, 95% CI: 46–98). Splenic origin was reported in 11 cases (8.7%, 95% CI: 3.8–14), abdominal involvement in 9 cases (7.1%, 95% CI: 2.1–11), while hepatic (2 cases, 1.6%, 95% CI: −0.59–3.7) and thyroidal localization (1 case, 0.8%, 95% CI: −0.75–2.3) were rare.

With respect to treatment patterns, the majority of patients received combined chemoimmunotherapy (114 cases, 89.8%, 95% CI: 84–95). Chemotherapy alone was administered to 7 patients (5.5%, 95% CI: 1.5–9.5), immunotherapy alone to 5 patients (3.9%, 95% CI: 0.55–7.33), and one patient (0.8%, 95% CI: 0–2.3) received a combination of chemotherapy, immunotherapy, and radiotherapy. Anthracycline-based regimens (doxorubicin) were given to 80 patients (63.0%, 95% CI: 55–71), while 47 patients (37.0%, 95% CI: 29–45) did not receive doxorubicin-containing protocols. The median cumulative doxorubicin dose at treatment completion was 250 mg/m^2^ (IQR = 410.0), with a maximum dose of 735 mg/m^2^. High-dose cyclophosphamide (≥150 mg/kg) was administered in 16 cases (12.6%, 95% CI: 6.8–18). Nearly all patients (122 cases, 96.1%, 95% CI: 93–99) received vincristine. The median number of chemotherapy cycles was 6.0 (IQR = 2.0), ranging from 1 to 8 cycles over a 6-month period.

### 3.2. Development of a Multimodal Model to Predict Cardiac Dysfunction Related to Antitumor Therapy in Non-Hodgkin Lymphoma

**Figure 2:**
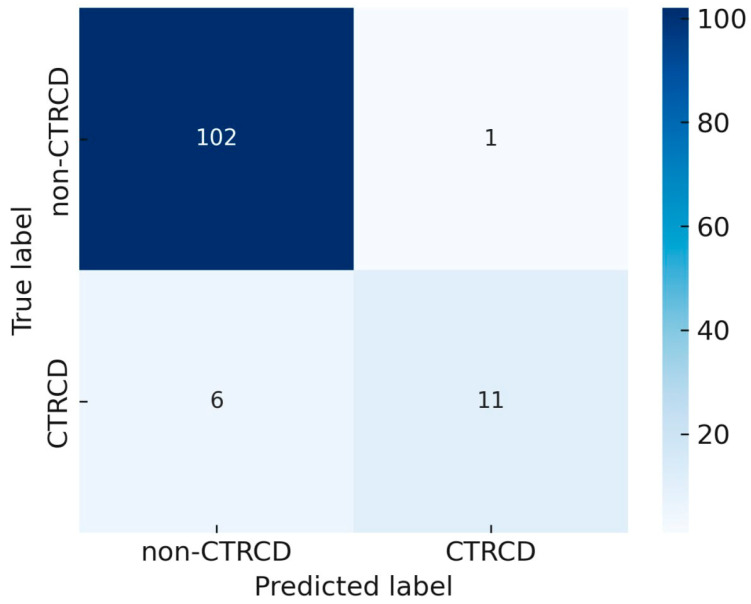
Assessment of the predictive accuracy of the model for identifying CTRCD.

To identify potential predictors, we selected parameters derived from the cardiovascular profile and cardiovascular risk factors, alongside serological markers, echocardiographic parameters, variables obtained from cardiopulmonary exercise testing, 24-h Holter ECG monitoring, and ambulatory blood pressure monitoring (ABPM). In addition, we considered parameters specific to non-Hodgkin lymphoma and its treatment. Within the cardiovascular risk profile, the assessed clinical indicators encompassed age, the presence of obesity (BMI > 30 kg/m^2^), active smoking, dyslipidemia, arterial hypertension, type 2 diabetes mellitus, chronic kidney disease, the calculated SCORE cardiovascular risk, as well as the Charlson comorbidity index. Indices obtained from 24-h Holter ECG or ABPM prior to initiation of antitumor therapy included: 24-h ABPM, SDNNi index, mean heart rate over 24 h, and mean corrected QT interval (QTc/24h). Echocardiographic parameters included: left atrial volume indexed to body surface area, left ventricular end-diastolic volume, left ventricular Tei index, E/A ratio, E/Vp ratio, E/e′ ratio, and right ventricular Tei index. The CPET parameters analyzed for their potential predictive value regarding cancer therapy-related cardiac dysfunction were: peak VO_2_ < 14 mL/kg/min, VO_2_ at the anaerobic threshold, and VO_2_ pulse. Serological parameters included: baseline hemoglobin, troponin I, and NT-proBNP levels. Treatment- and disease-related variables analyzed for their potential impact on CTRCD prediction after 6 months of therapy comprised: NHL stage, cumulative doxorubicin dose, rituximab administration, and chemotherapy regimens (COP, R-COP, R-CHOP, or other treatment schemes). When analyzing relative and absolute frequencies for predictive model development, it was observed that CTRCD occurred in 14.2% of patients. Since this rate exceeds 10%, standard classification methods could be applied for predictive modeling. The prediction model was developed using data from 120 respondents. Model performance in predicting 6-month cardiotoxicity was evaluated using a binary confusion matrix (2 × 2 table). The overall model accuracy was 94.2%. Specifically, the model correctly predicted the occurrence of cardiotoxicity in 11 patients who developed the complication, while it failed to predict CTRCD in 6 patients. Among patients who did not develop cardiotoxicity, 102 were correctly classified at 6-month follow-up, with only one false-positive case misclassified as developing therapy-related cardiac dysfunction when in fact it did not occur (Figure 2).

**Figure 3:**
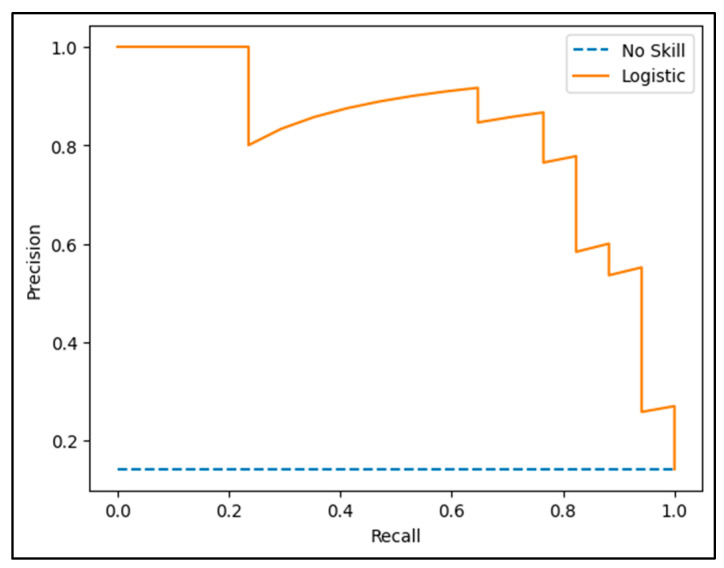
Performance analysis of the CTRCD prediction model through the precision-recall curve.

Figure 3 displays the precision-recall curve, illustrating the association between the true positive rate on the Y-axis and the false positive rate on the X-axis. A visual assessment of the curve shows a tendency to approach the upper left corner of the plot, suggesting a high level of discrimination. This indicates that the model successfully identifies the majority of true positive cases (patients who develop CTRCD) while minimizing misclassification of negative cases (patients who do not develop CTRCD). The area under the curve (AUC) value of 0.95, obtained from the predictive model analysis for cardiotoxicity, reflects strong performance in distinguishing between patients who subsequently develop cardiotoxicity and those who do not. This value indicates that the model can accurately detect 95% of positive cases (patients who experience cardiotoxicity) while considering false positive rates, highlighting its substantial clinical relevance. Moreover, an AUC of 0.95 signifies that, for any randomly selected pair of patients, the model has a 95% likelihood of assigning a higher risk score to the individual who will develop cardiotoxicity compared to one who will not. This finding demonstrates not only efficiency but also reliability, which is essential for supporting informed clinical decision-making.

**Figure 4:**
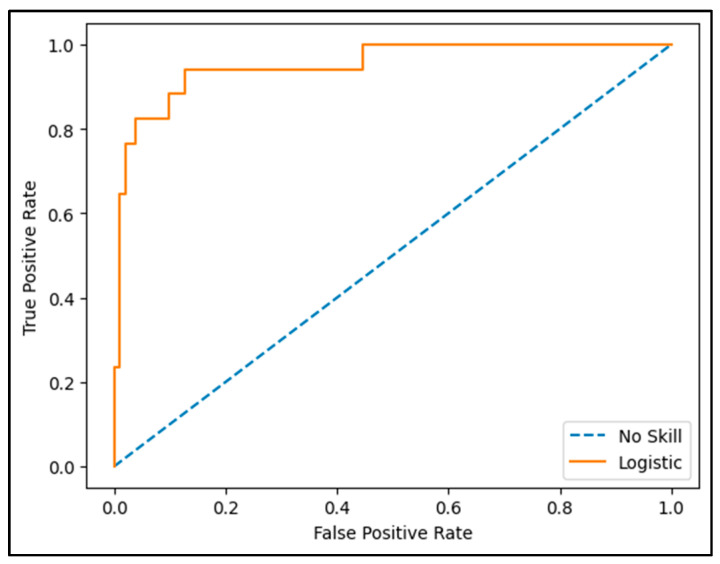
Evaluation of the accuracy and sensitivity of the CTRCD prediction model.

After the initial ROC curve assessment of the CTRCD prediction model, a further evaluation was carried out using precision and sensitivity metrics. This methodology offered an alternative perspective on model performance, enabling a more comprehensive appraisal of the trade-off between correctly identifying true positive cases and limiting misclassification errors. The ROC curve derived from this precision–sensitivity analysis demonstrated an AUC of 0.824, indicative of robust discriminative capability. These results suggest that the model reliably identifies patients at risk of developing cardiotoxicity within six months of therapy. Furthermore, an AUC of 0.824 indicates that, on average, the model correctly classifies patients with an 82.4% probability relative to random assignment (Figure 4).

**Figure 5:**
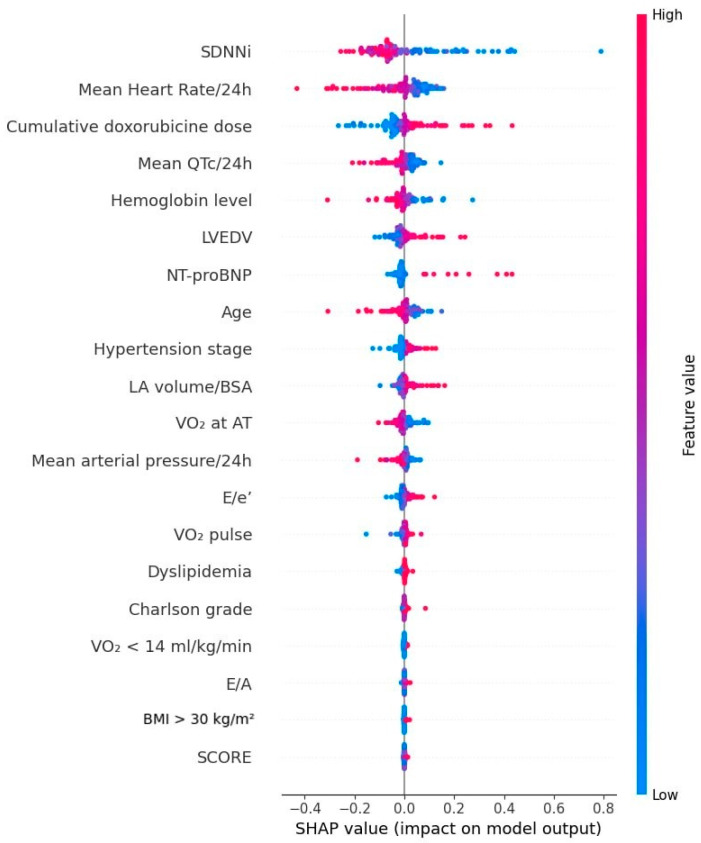
The contribution of clinical variables in the CTRCD prediction model.

Following the evaluation of model performance via ROC curves, an analysis based on additive explanations using Shapley values (SHAP analysis) was performed to gain a deeper understanding of each variable’s contribution to the cardiotoxicity prediction model. The resulting SHAP plot illustrates how each feature influences the model’s predictions. In Figure 5, SHAP values indicate the extent to which each component affects the predictive outcome. Baseline values from the first visit of SDNNi and mean heart rate obtained from 24-h Holter ECG exhibited the strongest influence on the model. Higher values of these parameters were associated with an increased predicted risk of subsequent CTRCD. Cumulative doxorubicin dose also emerged as a major contributing variable, with higher total doses substantially increasing predicted risk, reflecting the known association between anthracycline exposure and cardiotoxicity. Baseline hemoglobin levels had a considerable impact as well; lower hemoglobin values were associated with a reduced predicted risk, whereas higher values correlated with a less favorable prediction, potentially indicating patients with more complex medical conditions (e.g., compensatory polycythemia). Prolonged QTc intervals increased the likelihood of adverse outcomes, suggesting that QTc prolongation serves as an important prognostic marker in this model. Elevated baseline NT-proBNP levels were strongly associated with increased cardiotoxicity risk. Age, particularly higher values, was another significant predictor, contributing substantially to an increased predicted risk. Echocardiographic assessment highlighted left atrial volume indexed to body surface area and the E/e′ ratio as key determinants. Higher indexed left atrial volumes were moderately associated with an increased predicted risk. Elevated E/e′ ratios (red) were associated with a greater likelihood of developing therapy-induced cardiac dysfunction. The E/A ratio also contributed modestly, with higher values slightly increasing predicted risk, consistent with its association with more severe diastolic dysfunction or increased ventricular stiffness. Cardiopulmonary functional reserve, assessed by VO_2_ at the anaerobic threshold and peak VO_2_ < 14 mL/kg/min, provided insight into cardiopulmonary capacity, with lower values corresponding to higher predicted CTRCD risk, reflecting patients’ functional limitations.

**Figure 6:**
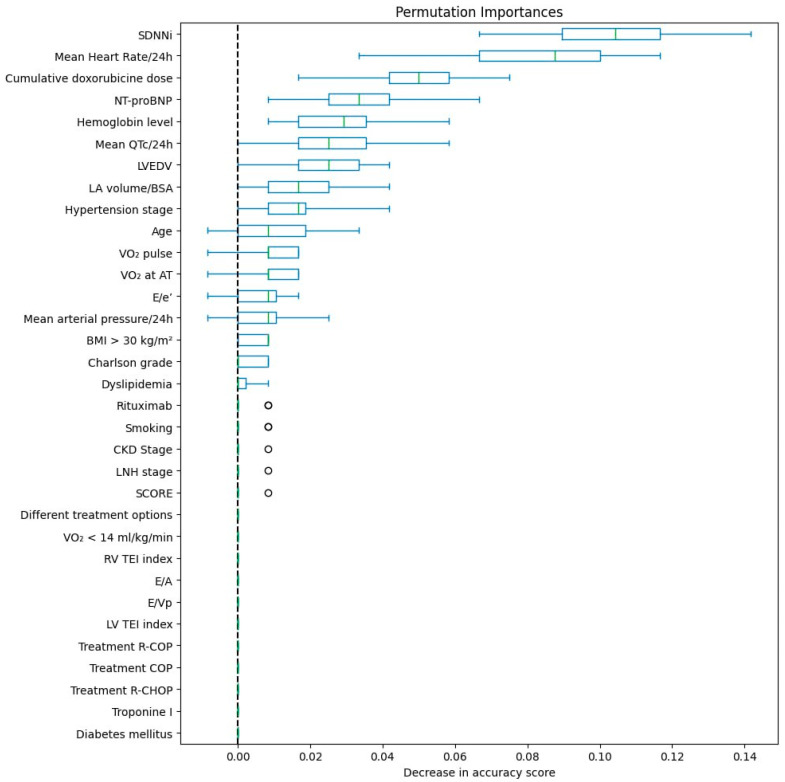
Impact of variables through permutation and SHAP mean values for the CTRCD prediction model.

The contribution of each variable to the model was evaluated by measuring the reduction in predictive accuracy following randomization or permutation of that variable’s values (Figure 6). Based on this analysis, SDNNi had the greatest numerical contribution to the model prediction (+0.12), with diminished heart rate variability strongly correlating with an elevated risk of developing CTRCD. Following SDNNi, the variables with the next highest influence were increased mean heart rate on 24-h Holter ECG and cumulative doxorubicin dose at six months, succeeded by baseline NT-proBNP, mean QTc over 24 h, and baseline hemoglobin, which demonstrated comparable effects on the model. In contrast, factors such as rituximab administration and various chemotherapy protocols (R-CHOP, COP, R-COP) showed lesser impact, indicating that although these variables may be clinically relevant in specific contexts, they are not among the strongest predictors of CTRCD within this predictive framework.

## 4. Discussion

Early identification of patients at high risk for CTRCD is essential to prevent irreversible cardiac dysfunction. In this regard, a number of studies have introduced multimodal predictive frameworks that combine clinical, imaging, and biometric parameters, with some employing artificial intelligence-based methodologies. One study proposed a CTRCD prediction model using clinical and echocardiographic variables [23]. Other analyses have focused on incorporating data from electrocardiograms. For example, one study demonstrated that artificial intelligence–based algorithms are capable of analyzing ECG recordings to detect subclinical cardiac alterations prior to the emergence of overt heart failure symptoms [14]. In a similar vein, another predictive model emphasized the value of baseline ECG assessments for estimating the risk of CTRCD in patients receiving anthracycline therapy [24]. These observations underscore the growing potential of non-invasive approaches in routine clinical practice. In patients with HER2-positive breast cancer, trastuzumab treatment is commonly linked to cardiotoxic events. A recent investigation proposed a personalized risk prediction model incorporating demographic and echocardiographic parameters, which can assist in optimizing therapeutic decision-making [25]. Additionally, heart rate variability has been explored as an early indicator of cardiotoxicity, with evidence suggesting that reductions in variability may precede measurable changes in left ventricular ejection fraction. Taken together, these studies highlight the substantial promise of multimodal predictive frameworks, particularly those enhanced by AI, for the early identification of CTRCD and the implementation of tailored cardiac monitoring strategies in oncology patients.

Other tools for evaluating heart failure risk aim to combine various clinical and demographic factors into an overall risk estimate; however, the best approach to predicting heart failure in cancer survivors remains unresolved. The HFA-ICOS score, developed by the Heart Failure Association in collaboration with the International Cardio-Oncology Society, is currently the only HF risk assessment tool specifically designed for adults with cancer. It incorporates variables related to cancer and its treatment, but since it was created for use at the start of chemotherapy, its effectiveness in predicting HF events in routine primary care or over long-term follow-up is still uncertain [26,27]. In general, non-cancer populations have relied on risk scores such as the ARIC-HF (Atherosclerosis Risk in Communities) and the modified Pooled Cohort Equations to Prevent Heart Failure (PCP-HF) to anticipate incident HF [26,27]; however, their accuracy and applicability in cancer survivor populations have not yet been fully determined. In comparative analyses, both HFA-ICOS and ARIC-HF demonstrated superior performance relative to PCP-HF across cancer and non-cancer cohorts, although all three instruments exhibited only modest discriminative ability for incident heart failure in clinical practice. This highlights the potential value of a cancer-specific heart failure risk assessment tool, which could improve early identification of high-risk individuals and support targeted preventive strategies among cancer survivors [28].

In our study, the model integrated variables from clinical, serological, and instrumental diagnostic domains. The model was constructed employing SHAP analysis, which prioritizes variables based on their relative contribution to the model’s performance. Such an approach can be incorporated into existing medical systems to automatically estimate patient status regarding the risk of therapy-related cardiac dysfunction, potentially guiding clinical interventions. The model’s multivariate design effectively supports binary prediction, determining the potential occurrence of the adverse event. Its clinical integration allows early identification of patients undergoing antitumor therapy who may develop CTRCD. This strategy enables early and targeted intervention, optimizing both clinical monitoring and resource allocation.

## 5. Limitations

The study may be affected by unaccounted interactions between different chemotherapy regimens due to the limited sample size, which could influence outcomes. Additionally, the impact of cardiovascular risk factors on CTRCD development might be underestimated. The lack of comparison with healthy controls or non-CTRCD patients limits the contextual understanding of therapy-related cardiac effects. Future studies with larger cohorts and stratified analyses are needed to address these issues. One limitation of this study is the moderate class imbalance between patients with and without CTRCD (18 of 127, ≈14%). Although this proportion does not represent an extreme imbalance (<10%), it may still bias model performance. The limited number of positive cases can restrict the model’s ability to learn discriminative patterns for CTRCD and may lead to optimistic overall accuracy but reduced sensitivity for the minority class.

## Figures and Tables

**Table 1:** Baseline demographic and clinical data for patients with/without CTRCD.

	Group I (CTRCD, *n* = 18) ^1^	CI ^2^ 95%	Group II (Without CTRCD, *n* = 109) ^1^	CI ^2^ 95%	Statistic Test	*p* ^3^
Age (years)	65.3 (11.9) 69.5 (16.5) 35.0, 83.0	59, 71	60.9 (10.0) 62.0 (13.0) 34.0, 80.0	59, 63	1243	0.071
Sex					1.3	0.3
Male	8 (44.4)	21, 67	64 (58.7)	49, 68		
Female	10 (55.6)	33, 79	45 (41.3)	32, 51		
BMI (kg/m^2^)	28.2 (5.1) 28.7 (8.3) 18.6, 36.8	26, 31	26.6 (5.6) 25.2 (6.6) 19.2, 48.7	26, 28	1217	0.10
BMI > 30 kg/m^2^	8 (44.4)	21, 67	23 (21.1)	13, 29	4.6	**0.043**
AC (cm)	88.7 (20.7) 93.5 (33.8) 59.0, 124.0	78, 99	83.9 (17.8) 78.0 (20.0) 57.0, 139.0	80, 87	1108	0.4
Worsened anamnesis	7 (38.9)	16, 61	16 (14.7)	8.0, 21	6.1	**0.022**
Smoking	4 (22.2)	3.0, 41	26 (23.9)	16, 32	0.02	>0.9
Hypertension grade					24	**<0.001**
gr.I	2 (11.1)	−3.4, 26	2 (1.8)	−0.68, 4.4		
gr.II	6 (33.3)	12, 55	29 (26.6)	18, 35		
gr.III	6 (33.3)	12, 55	5 (4.6)	0.66, 8.5		
Diabetes mellites	5 (27.8)	7.1, 48	13 (11.9)	5.8 18	3.2	0.13
CKD					9.0	**0.029**
CKD G1/2	2 (11.1)	−3.4, 26	12 (11.0)	5.1, 17		
CKD G3	3 (16.7)	−0.55, 34	2 (1.8)	−0.68, 4.4		
Chronic bronchitis	5 (27.8)	7.1, 48	22 (20.2)	13, 28	0.53	0.5
Thyroid pathology	1 (5.6)	−5.0, 16	8 (7.3)	2.4, 12	0.07	>0.9

Note: ^1^ mean (SD), median (IQR), minimum, maximum; *n* (%) ^2^ confidence interval (CI), ^3^ Kruskal–Wallis test, Pearson Chi-Square test with estimated *p*-value.

## Data Availability

The data presented in this study are available on request from the corresponding author.

## Abbreviations

The following abbreviations are used in this manuscript:

ABPM	ambulatory blood pressure monitoring
ARIC-HF	Atherosclerosis Risk in Communities—Heart Failure
AUC	area under the curve
BMI	body mass index
CI	confidence interval
CKD	chronic kidney disease
CO_2_	carbon dioxide
COP	cyclophosphamide, vincristine, prednisone
CPET	cardiopulmonary exercise testing
CTRCD	cancer therapy-related cardiac dysfunction
ECG	electrocardiogram
HER2	human epidermal growth factor receptor 2
HFA-ICOS	Heart Failure Association—International Cardio-Oncology Society
IQR	interquartile range
LVEF	left ventricular ejection fraction
NHL	non-Hodgkin lymphoma
OUES	oxygen uptake efficiency slope
PCP-HF	Prediction of Cardiovascular events in Primary Care—Heart Failure
R-CHOP	rituximab, cyclophosphamide, doxorubicin, vincristine, prednisone
R-COP	rituximab, cyclophosphamide, vincristine, prednisone
RER	respiratory exchange ratio
ROC	receiver operating characteristic curve
SDANN	standard deviation of the averages of NN intervals
SDNN	standard deviation of all normal-to-normal intervals
SDNNi	standard deviation of the normal-to-normal intervals index
SHAP	Shapley additive exPlanations analysis
VCO_2_	volume of carbon dioxide
VE/VCO_2_	ventilatory equivalent for carbon dioxide
VO_2_	volume of oxygen
VO_2_ peak	peak oxygen uptake
VO_2_ pulse	volume of oxygen per heart rate
VO_2_/WR	volume of oxygen per work rate
